# Inadequate immune response to inactivated COVID-19 vaccine among older people living with HIV: a prospective cohort study

**DOI:** 10.1128/jvi.00688-25

**Published:** 2025-08-21

**Authors:** Hangjie Zhang, Xuan Deng, Rongrong Dai, Jian Fu, Lingling Ding, Xiaowei Hu, Pinjing Sun, Renping Shu, Lifeng Chen, Xiaoping Xu

**Affiliations:** 1 Department of Prevention and Control of Infectious Disease, Key Lab of Vaccine, Zhejiang Provincial Center for Disease Control and Prevention 117838, Hangzhou, China; 2 School of Public Health, Hangzhou Medical College 117839, Hangzhou, China; 3 Department of Immunization Program, Zhejiang Provincial Center for Disease Control and Prevention 117838, Hangzhou, China; 4 Xihu District Center for Disease Control and Prevention, Hangzhou, China; 5 Jiande County Center for Disease Control and Prevention, Hangzhou, China; 6 Haining County Center for Disease Control and Prevention, Jiaxing, China; 7 Yuyao City Center for Disease Control and Prevention, Ningbo, China; University of North Carolina at Chapel Hill, Chapel Hill, North Carolina, USA

**Keywords:** inactivated COVID-19 vaccine, older people, PLWH, safety, immunogenicity

## Abstract

The concerns exist regarding the inadequate immune response to inactivated COVID-19 vaccines in some people living with HIV (PLWH). A prospective cohort study was conducted across four regions of Zhejiang, China. Both PLWH and healthy controls (HCs) received two doses of the inactivated COVID-19 vaccine. Adverse reactions within 7 days after each injection were monitored. PLWH with CD4 counts <500 cells/µL received a third dose. Neutralizing antibodies (NAbs) and IgG were measured to evaluate the immunogenicity of each dose. A total of 400 PLWH were enrolled, with 12.2% aged ≥60 years. Adverse reactions to the first two doses were mild or moderate in PLWH (12.0% and 5.5%). After the second dose, the geometric mean titer (GMT) of NAbs was lower in PLWH (46.9, 95% confidence interval [CI] 43.6–50.4) compared to HCs (58.2, 95% CI 53.4–63.5). However, seropositivity rates were similar (97.9% vs 100.0%). Single and multifactor generalized estimating equation analysis revealed that PLWH aged ≥60 years with CD4 count <500 cells/µL exhibited a weaker immune response. After a third dose in this subgroup (aged ≥60 years), the GMT of NAbs was 61.5 (95% CI 47.8–79.2), significantly lower than the 18–59 years group. In conclusion, PLWH showed a weaker and delayed immune response after receiving two doses of the inactivated COVID-19 vaccine. Older age combined with a lower CD4+ T cell count posed significant challenges to achieving an adequate immune response. Strategies to improve COVID-19 vaccine uptake and overall immunization in PLWH are needed.

**IMPORTANCE:**

In this prospective cohort study, we analyzed the safety and immunogenicity of a two-dose schedule of the inactivated COVID-19 vaccine (Covilo) in people living with HIV (PLWH). We also investigated factors associated with antibody titers and seropositivity rates. Additionally, we evaluated the immunogenicity of an inactivated COVID-19 vaccine booster in PLWH with CD4+ T cell counts ≤500 cells/µL within both 18–59 and ≥60 years age groups. We found vaccination with the inactivated COVID-19 vaccine was well-tolerated in PLWH but induced a weaker and delayed immune response compared with healthy controls. Importantly, the older PLWH with low CD4+ T cell counts face considerable challenges in mounting an adequate immune response to vaccines, even after booster immunization. Our findings highlight the need for strategies to improve vaccine immunogenicity in PLWH with low immune responses, particularly among older individuals and those with low CD4+ T cell counts.

**CLINICAL TRIALS:**

This study is registered with ClinicalTrials.gov as NCT05075070.

## INTRODUCTION

Human immunodeficiency virus (HIV), first identified in 1983 as the cause of acquired immunodeficiency syndrome (AIDS), is now a treatable condition with the potential for long-term survival (1, 2). However, people living with HIV (PLWH) remain at increased risk of illness and death owing to progressive CD4+ T cell depletion and chronic immune activation, leaving them vulnerable to opportunistic infections (3). The World Health Organization estimates that approximately 39 million people worldwide live with HIV, with an estimated 2 million new infections occurring annually (4).

The ongoing coronavirus disease 2019 (COVID-19) pandemic has further amplified the risks for PLWH; they are more likely to experience severe illness and worse outcomes during HIV progression (5). Current COVID-19 vaccines are widely used; they have demonstrated remarkable safety and efficacy in preventing infection and reducing disease severity (6–8). Consequently, PLWH have been prioritized for COVID-19 vaccination, with primary and booster schedules recommended based on CD4+ T cell count and HIV viremia levels in China and other regions (9–11). Notably, PLWH with residual inflammation on antiretroviral therapy (ART) and compromised immune systems may experience a higher incidence of vaccine adverse events and a reduced response to vaccination (12–14).

CD4+ T cells served as a reservoir for HIV, with increased antigenic and inflammatory loads during infection leading to greater immune activation and exhaustion in PLWH compared to healthy controls (15). This causes CD4+ T cell exhaustion and clonal depletion, which ultimately leads to a decline in immune cell proliferation, a gradual deterioration of immunological memory, and an impact on the long-lasting and protective antibody levels following vaccination (16). Although ART suppresses viral replication and partially restores immune function in PLWH, persistent CD4+ T cell depletion remains common, causing progressive immune system failure.

Previous studies have shown that vaccination with meningococcal serogroup B (4CMenB) vaccine (17), yellow fever vaccine (18), and influenza A (H1N1) vaccine (19) is effective against infection in PLWH, but CD4+ cell counts influenced vaccine response, with higher counts (>200 cells/mm^3^) associated with better responses (12). The lower seroconversion rates to Hepatitis B virus vaccine were observed in PLWH with low CD4+ T cell levels (20). Several studies about COVID-19 vaccines have shown that PLWH with CD4+ T cell counts ≤500 cells/µL (21), ≤350 cells/µL (22), and especially those with <200 cells/µL, exhibit lower severe acute respiratory syndrome coronavirus 2 (SARS-CoV-2) IgG and neutralizing antibody (NAbs) titers and seropositivity rates relative to PLWH with higher CD4+ T cell counts after COVID-19 vaccination. Furthermore, the frequencies of RBD-specific memory B cells and interferon-γ/tumor necrosis factor-α secreting CD4+ T cells are reduced in PLWH with CD4+ T cell counts <200 cells/µL (14, 23). These findings suggest that the diminished immune response after inactivated COVID-19 vaccination in PLWH largely results from CD4+ T cell depletion and immunosuppression.

Our previous study showed that healthy older adults tend to have a weaker immune response than younger adults after receiving inactivated COVID-19 or recombinant protein subunit vaccines (ZF2001) (24, 25). The availability of combination ART has significantly increased life expectancy in the HIV-infected population, leading to a growing number of older PLWH (26). However, few studies have focused on the immune response to COVID-19 vaccines in this population. Zeng et al. reported that younger PLWH (aged 8–39 years) produced higher levels of RBD IgG antibodies than those aged ≥40 years after three doses of an inactivated COVID-19 vaccine (27). Conversely, other studies have shown that age does not influence NAbs seroconversion rates in multivariable models (21, 28).

In this prospective cohort study, we analyzed the safety and immunogenicity of a two-dose schedule of the inactivated COVID-19 vaccine (Covilo) in PLWH. We also investigated factors associated with antibody titers and seropositivity rates. Additionally, we evaluated the immunogenicity of an inactivated COVID-19 vaccine booster in PLWH with CD4+ T cell counts ≤500 cells/µL within both 18–59 and ≥60 years age groups. Our findings highlight the need for strategies to improve vaccine immunogenicity in PLWH with low immune responses, particularly among older individuals and those with low CD4+ T cell counts.

## MATERIALS AND METHODS

### Study design and participants

This multicenter prospective cohort study was conducted in four districts (Xihu District, Yuyao City, Jiande County, and Haining County) of Zhejiang province, China, from August 2021 to May 2022. HIV-infected patients from the China National HIV/AIDS Comprehensive Response Information Management System (CRIMS) and healthy controls from the community were invited to participate.

Inclusion criteria were (i) age 18–80 years and (ii) willingness to receive inactivated COVID-19 vaccines. Exclusion criteria were (i) history of SARS-CoV-2 infection or active infection at vaccination (confirmed by serological and nucleic acid tests), (ii) history of allergic reaction to vaccine components, (iii) severe acute respiratory disease or active infectious disease within the preceding month, and (iv) ongoing immunosuppressive drug treatment. Demographic, clinical, and laboratory data regarding HIV status were obtained from the CRIMS.

### Procedures

For phase 1, eligible participants received two doses of Covilo in May 2022, 21 days apart. After each vaccination, participants were monitored for 30 minutes for immediate adverse reactions and asked to record any events in a diary card for the next 7 days. Self-reported serious adverse events were documented throughout the study. Peripheral venous blood samples (10 mL each) were collected at baseline (T0), 28 days (T1), and 3–6 months (T2) after the second dose. For phase 2, at 3 months after the second dose, 64 PLWH with CD4 <500 cells/µL (aged 18–59 and ≥60 years, 1:1 ratio) received a booster dose of Covilo. Blood samples (10 mL) were collected 28 days (T3) after the booster dose. These samples were used to assess SARS-CoV-2 NAbs and SARS-CoV-2-specific IgG antibody levels.

### Safety assessments

The safety profile of the Covilo vaccine was assessed by recording any solicited local and systemic reactions on diary cards within 7 days after the first and second doses. Solicited local reactions included itching, pain, induration, redness, swelling, and rashes; systemic reactions included coughing, diarrhea, headache, fatigue, fever, myalgia, nausea, and vomiting.

### Microneutralization assays

The titer of NAbs to live SARS-CoV-2 was measured by the reduction in cytopathic effect on Vero cells infected with the SARS-CoV-2 strain 19nCoVCDC-Tan-HB01 in a BSL-3 laboratory (29). Briefly, serum samples were heat-inactivated at 56°C for 30 min, then serially diluted in 96-well plates. The diluted samples were incubated with an equal volume of challenge solution containing 100 TCID50 (50% tissue culture infectious dose) of the virus for 2 hours at 37°C. A cell suspension (1.5–2.5 × 10^5^ cells/mL) was added to each well. The cytopathic effect was analyzed at 4 days post-infection. The neutralization titer was expressed as the NT50 (reciprocal of the highest dilution protecting 50% of cells from the virus). An NT50 above 1:8 was considered a positive result.

### Binding antibody assay

Binding antibodies were measured using two commercial assays (30). Serum levels of IgG against SARS-CoV-2 spike (S) and nucleocapsid (N) proteins were determined by chemiluminescence immunoassay using the 2019-nCoV kit (iFlash, Shenzhen, China). Briefly, serum samples were incubated with S- and N-protein antigen-coated paramagnetic microparticles. An acridinium-ester-labeled ACE2 conjugate was then added to compete for binding to the particles, forming a reaction mixture. The analyzer converted relative light units into antibody titers (AU/mL) using a two-point calibration curve. IgG against the SARS-CoV-2 RBD was detected by enzyme-linked immunosorbent assays using commercial kits (Bioscience Biotech Co. Ltd., Chongqing, China). According to the manufacturer, anti-S&N IgG concentrations ≥10.0 AU/mL and anti-RBD IgG concentrations ≥1.0 AU/mL were considered positive results.

### Statistical analysis

Sex, age, CD4 counts, and other clinical characteristics were collected for each vaccine recipient. We used medians and interquartile range (IQR) to report age, numbers (percentages) for categorical variables, and means ± standard deviations for continuous variables. All participants who received each dose were included in the safety population (i.e., safety set). Safety data are presented as counts and percentages of participants experiencing at least one solicited (local or systemic) reaction. Immunogenicity outcomes are reported based on the per-protocol set, with no imputation for missing data. Specific binding antibodies against SARS-CoV-2 (IgG) and the neutralized fraction of SARS-CoV-2 NAbs are presented as the geometric mean concentration (GMC)/geometric mean titer (GMT), seroconversion/seropositivity rate, and GMFI with 95% confidence intervals. We used Student’s *t*-test and one-way analysis of variance with Tukey’s multiple comparisons test to analyze log-transformed antibody titers and categorical data. The Mann–Whitney U test and Wilcoxon rank-sum test were utilized for non-normally distributed data. The chi-square test and Fisher’s exact test were performed to analyze categorical data. A logistic regression model (enter method) was used for multivariate analysis. Correlation methods were implemented for bivariate analysis, and multiple linear regression was conducted to evaluate independent risk factors. *P* values <0.05 were considered statistically significant (*P < 0.05; **P < 0.01, ***P < 0.001, ****P < 0.0001). All statistical analyses were conducted using SPSS 18.0 (IBM Corporation, Armonk, NY, USA), R (version 4.2.1), and GraphPad Prism 9 (San Diego, CA, USA) software.

## RESULTS

### Characteristics of enrolled individuals

**Fig 1:**
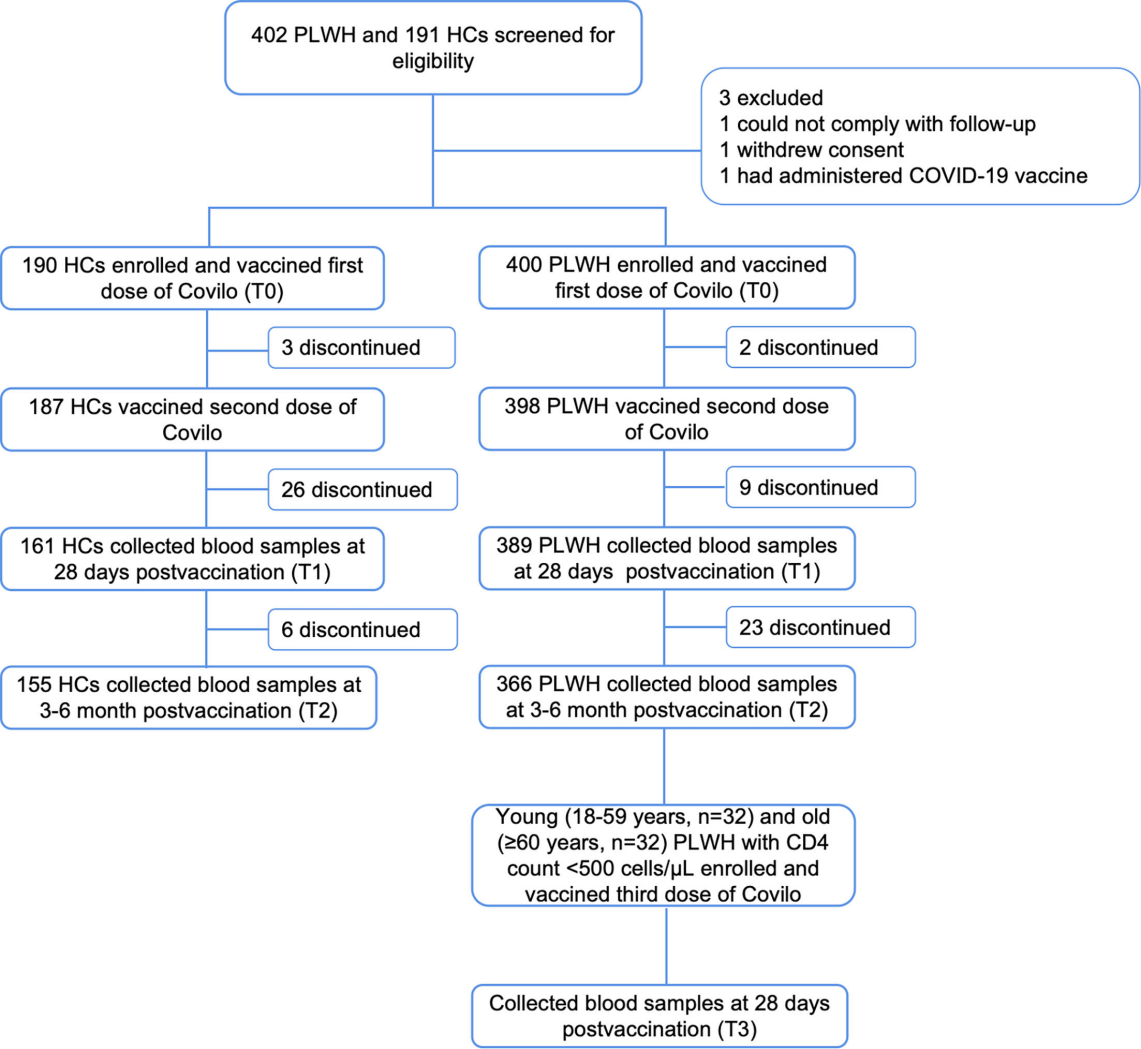
Study profile for the vaccination schedule and follow-up. PLWH and HCs were screened by inclusion and exclusion criteria, and the enrolled subjects received two doses of Covilo 21 days apart. The adverse reactions were documented within 7 days of the first and second dose. Peripheral venous blood samples were collected at baseline (T0), 28 days (T1), and 3–6 months (T2) after the second dose for antibody detection. Young (18–59 years, *n* = 32) and old (≥60 years, *n* = 32) PLWH with CD4 count <500 cells/µL enrolled and vaccinated third dose of Covilo, also collected blood samples for antibody detection at 28 days post-vaccination (T3).

In total, 400 PLWH and 190 healthy controls (HCs) were enrolled and received two doses of the inactivated COVID-19 vaccine, Covilo. The median age of PLWH was 36 years (IQR, 29–51 years); 59.0% were aged 18–39 years, 28.8% were aged 40–59 years, and 12.2% were aged ≥60 years. The majority of PLWH (92.5%) were men (Table 1). All PLWH were on stable ART (27.0% for <2 years, 25.0% for 2–4 years, and 48.0% for ≥4 years) prior to the first dose, 89.3% (357/400) had an HIV RNA viral load <50 copies/mL. The mean CD4 count was 433 ± 241.8 cells/µL, and 34.2% (137/400) had CD4+ T cell counts above 500 cells/µL, with 15.0% (60/400) PLWH having CD4+ T cell counts <200 cells/µL. The median age of HCs was 63 years (IQR, 50–70 years), and 46.3% were men. The baseline characteristics of all participants are shown in Table 1. All participants received two doses of Covilo and completed a 6-month post-vaccination follow-up, during which 34 PLWH and 35 HCs were lost to follow-up (Fig. 1). Additionally, 64 PLWH with CD4 counts <500 cells/µL (32 aged 18–59 years and 32 aged ≥60 years) received a third booster dose.

**TABLE 1:** Baseline characteristics^*a*^

Variable	PLWH (*n* = 400)	HCs (*n* = 190)	*P* value
Age, years			<0.001
18–39	236 (59.0)	33 (17.4)	
40–59	115 (28.8)	50 (26.3)	
≥60	49 (12.2)	107 (56.3)	
Median (IQR)	36.0 (29.0, 50.0)	62.5 (50.0, 70.3)	
Sex			<0.001
Male	370 (92.5)	88 (46.3)	
Female	30 (7.5)	102 (53.7)	
HIV-related health conditions^*b*^			N.A.^*e*^
Tuberculosis	3 (0.8)	N.A.	
Cryptococcal pneumonia	1 (0.3)	N.A.	
Oral inflammation or ulcers	6 (1.5)	N.A.	
Generalized pruritic dermatitis	1 (0.3)	N.A.	
Diarrhea	4 (1.0)	N.A.	
CD4+ T cell counts, cells/µL, n (%)			N.A.
<200	60 (15.0)	N.A.	
200–500	203 (50.8)	N.A.	
≥500	137 (34.2)	N.A.	
Mean (±SD)	443.2 (241.8)	N.A.	
HIV viral loads before the first dose of vaccine, copies/mL^*c*^	4,382 (228, 22,850)	N.A.	N.A.
<50	357 (89.3)	N.A.	
≥50	20 (5.0)	N.A.	
Unknown	23 (5.7)	N.A.	
Years of starting ART			N.A.
<2	108 (27.0)	N.A.	
2–4	100 (25.0)	N.A.	
≥4	192 (48.0)	N.A.	
ART use, n (%)			N.A.
3TC+EFV+TDF	183 (45.8)	N.A.	
3TC+EFV+AZT	63 (15.8)	N.A.	
ECFTAF	50 (12.5)	N.A.	
3TC+DTG+TDF	26 (6.5)	N.A.	
3TC+ABC+DTG	10 (2.5)	N.A.	
3TC+AZT+NVP	10 (2.5)	N.A.	
Other^*d*^	58 (14.5)	N.A.	

^*a*^ Data are presented as the number of participants (%), mean (SD), or median (IQR). Abbreviations: 3TC, lamivudine; ABC, abacavir; ART: antiretroviral therapy; AZT, Zidovudine; DTG, dolutegravir; ECFTAF: Elvitegravir, Cobicistat, Emtricitabine, and Tenofovir Alafenamide Fumarate tablets; EFV, efavirenz; NVP, nevirapine; TDF, tenofovir disoproxil fumarate.

^*b*^ HIV-related health conditions “weight loss, fever, pneumocystis carinii pneumonia, cytomegalovirus infection, diarrhea, tuberculosis, oral inflammation or ulcers, novel cryptococcal meningitis/cryptococcal pneumonia, recurrent bacterial pneumonia, persistent generalized lymph node enlargement, herpes simplex or chronic recurrent episodes of herpes zoster, lymphoma, mucosal or visceral Kaposi's sarcoma, generalized pruritic dermatitis”.

^*c*^ The data presented in PLWH with a positive HIV viral load.

^*d*^ Others contain unclear information about the regimen and other regimens which were not displayed.

^*e*^ N.A., not applicable.

### Safety outcomes

Vaccine-related adverse events are detailed in Table 2 and Table S1. Within 7 days of the first dose, 48 PLWH (12.0%) reported adverse reactions, including 22 (5.5%) local and 33 (8.3%) systemic reactions, whereas only five HCs (2.6%) experienced adverse events. The most common adverse reactions after the first dose in PLWH were injection site pain (3.5%), followed by headache (2.8%), myalgia (2.5%), and fatigue (2.0%). In HCs, injection site pain was the most common adverse event (1.1%). There was no increase in the total number of adverse reactions reported after the second dose (22 [5.5%] in PLWH and four [2.1%] in HCs). The most common reactions after the second dose were similar to the first, primarily injection site pain and fatigue. Fewer adverse reactions were observed in PLWH with CD4 counts <200 cells/µL (4 [6.7%]) relative to those with CD4 counts of 200–500 cells/µL (11.8%) and ≥500 cells/µL (14.6%). All adverse reactions in both groups were mild, transient, self-limiting, and required no treatment. No vaccine-related severe adverse events were observed in either group.

**TABLE 2:** Adverse reactions within 7 days after the first and second dose of vaccine^*a*^

Variable	PLWH				HCs
	Total	CD4 < 200	CD4 (200–500)	CD4 ≥ 500	
First dose					
Total adverse reactions	48 (12.0)	4 (6.7)	24 (11.8)	20 (14.6)	5 (2.6)
Local reactions	22 (5.5)	2 (3.3)	12 (5.9)	8 (5.8)	3 (1.6)
Itching	1 (0.3)	0 (0)	0 (0)	1 (0.7)	0 (0)
Pain	16 (4.0)	2 (3.3)	5 (2.5)	9 (6.6)	2 (1.1)
Induration	0 (0)	0 (0)	0 (0)	0 (0)	0 (0)
Redness	0 (0)	0 (0)	0 (0)	0 (0)	0 (0)
Swelling	1 (0.3)	0 (0)	1 (0.5)	0 (0)	1 (0.5)
Rash	5 (1.3)	0 (0)	2 (1)	3 (2.2)	0 (0)
Systemic reactions	33 (8.3)	3 (5)	17 (8.4)	13 (9.5)	2 (1.1)
Coughing	1 (0.3)	0 (0)	0 (0)	1 (0.7)	0 (0)
Diarrhea	7 (1.8)	2 (3.3)	3 (1.5)	2 (1.5)	0 (0)
Fatigue	8 (2)	2 (3.3)	2 (1)	4 (2.9)	0 (0)
Fever	2 (0.5)	0 (0)	1 (0.5)	1 (0.7)	0 (0)
Headache	11 (2.8)	1 (1.7)	6 (3)	4 (2.9)	1 (0.5)
Myalgia	10 (2.5)	0 (0)	6 (3)	4 (2.9)	0 (0)
Nausea and vomiting	5 (1.3)	0 (0)	2 (1)	3 (2.2)	1 (0.5)
Second dose					
Total adverse reactions	22 (5.5)	2 (3.4)	14 (6.9)	6 (4.4)	4 (2.1)
Local reactions	16 (4)	2 (3.4)	9 (4.5)	5 (3.6)	2 (1.1)
Itching	0 (0)	0 (0)	0 (0)	0 (0)	0 (0)
Pain	15 (3.8)	1 (1.7)	9 (4.5)	5 (3.6)	2 (1.1)
Induration	0 (0)	0 (0)	0 (0)	0 (0)	0 (0)
Redness	0 (0)	0 (0)	0 (0)	0 (0)	0 (0)
Swelling	1 (0.3)	0 (0)	1 (0.5)	0 (0)	0 (0)
Rash	2 (0.5)	1 (1.7)	1 (0.5)	0 (0)	0 (0)
Systemic reactions	9 (2.3)	0 (0)	8 (4)	1 (0.7)	3 (1.6)
Coughing	1 (0.3)	0 (0)	1 (0.5)	0 (0)	0 (0)
Diarrhea	1 (0.3)	0 (0)	1 (0.5)	0 (0)	0 (0)
Fatigue	7 (1.8)	0 (0)	6 (3)	1 (0.7)	3 (1.6)
Fever	0 (0)	0 (0)	0 (0)	0 (0)	0 (0)
Headache	0 (0)	0 (0)	0 (0)	0 (0)	0 (0)
Myalgia	1 (0.3)	0 (0)	1 (0.5)	0 (0)	0 (0)
Nausea and vomiting	0 (0)	0 (0)	0 (0)	0 (0)	0 (0)

^*a*^ Data are presented as the number of participants experiencing the event (%). Each participant was counted only once within each specific reaction category, even if they experienced multiple adverse reactions. The "Total" category includes all participants who experienced adverse reactions or events of any grade. Adverse reactions and events were graded according to the scale issued by the China State Food and Drug Administration.

### Immunogenicity assessment

**Fig 2:**
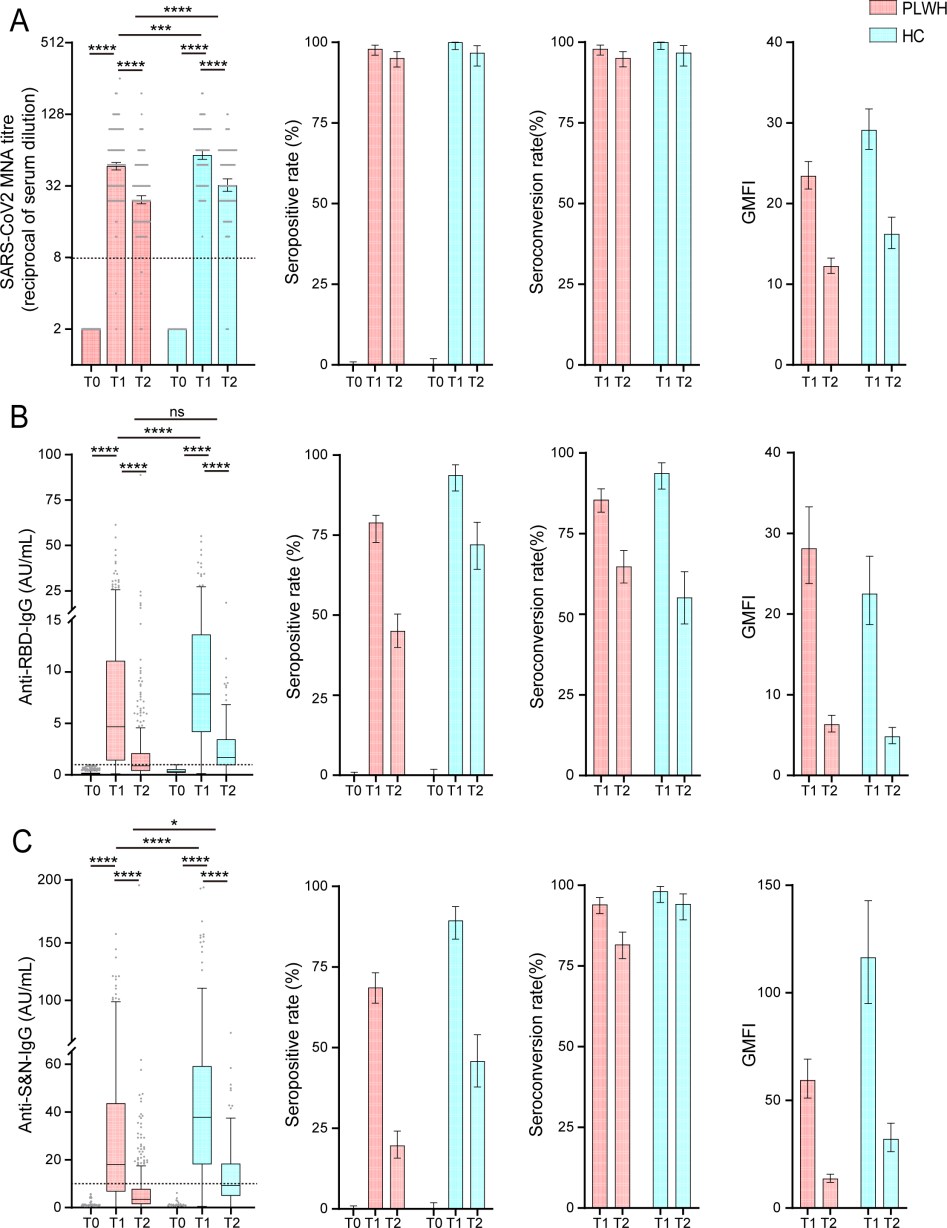
Antibody responses before and after two doses of vaccine. GMT/GMC, seropositivity rates (%), seroconversion rates (%), and GMFI of NAbs (**A**), anti-RBD IgG (**B**), and anti-S&N IgG (**C**) to SARS-CoV-2 (HB01) at baseline (T0), 28 days (T1), and 3–6 months (T2) after the second dose of Covilo in PLWH and HCs. Data are presented as geometric mean ± 95% CI or as box and whisker plots, indicating median (middle line), 25th, and 75th percentiles (box). Seroconversion was defined as at least a fourfold increase in antibody titers at T1 and T2 relative to T0. Statistical significance was determined by two-tailed Student’s *t*-test and one-way analysis of variance (ANOVA) with Tukey’s multiple comparisons test. *P* values <0.05 were considered statistically significant. 95% CI, 95% confidence interval; GMT, geometric mean titer; GMC, geometric mean concentration; GMFI, geometric mean fold increase; MNA, microneutralization assays. ns, no significance; *P < 0.05, **P < 0.01, ***P < 0.001, ****P < 0.0001.

No participants had detectable NAbs or IgG against SARS-CoV-2 at baseline (T0). At 28 days after the second vaccination (T1), the GMT of NAbs was significantly lower in PLWH than in HCs (46.9 [95% CI 43.6–50.4] vs 58.2 [95% CI 53.4–63.5]; *P* < 0.001), although the seropositivity and seroconversion rates were similar (97.9% vs 100.0%) (Fig. 1 and Table 2). Additionally, the GMFIs of NAbs from pre-immunization were 23.5 (95% CI 21.8–25.2) in PLWH and 29.1 (95% CI 26.7–31.7) in HCs. The GMCs of anti-RBD and anti-S&N IgG were also lower in PLWH than in HCs (3.8 AU/mL [95% CI 3.3–4.4] vs 7.1 AU/mL [95% CI 6.0–8.4] and 15.0 AU/mL [95% CI 13.1–17.2] vs 31.2 AU/mL [95% CI 26.4–36.7]; *P* < 0.001 for both). Seropositivity rates showed similar trends: 78.9% (95% CI 72.7–81.2) vs 93.8% (95% CI 88.8–97.0) for anti-RBD IgG and 68.6% (95% CI 63.8–73.2) vs 89.4% (95% CI 83.6–93.7) for anti-S&N IgG (Table 3 and Fig. 2).

**TABLE 3:** RBD-specific IgG, S&N-specific IgG, and neutralizing antibody^*a*^

Variable	PLWH			HCs		
	T0 (*n* = 400)	T1 (*n* = 389)	T2 (*n* = 366)	T0 (*n* = 190)	T1 (*n* = 161)	T2 (*n* = 155)
Neutralizing antibody						
GMT	2.0 (2.0–2.0)	46.9 (43.6–50.4)	24.5 (22.7–26.5)	2.0 (2.0–2.0)	58.2 (53.4–63.5)	32.5 (28.8–36.6)
Seropositive rate (%)	0.0 (0.0–0.9)	97.9 (96.0–99.1)	95.1 (92.3–97.1)	0.0 (0.0–1.9)	100.0 (97.7–100.0)	96.8 (92.6–98.9)
Seroconversion rate (%)	N.A.	97.9 (96.0–99.1)	95.1 (92.3–97.1)	N.A.	100.0 (97.7–100.0)	96.8 (92.6–98.9)
GMFI	N.A.	23.5 (21.8–25.2)	12.3 (11.3–13.2)	N.A.	29.1 (26.7–31.7)	16.2 (14.4–18.3)
Anti-RBD-IgG						
GMC (AU/mL)	0.1 (0.1–0.1)	3.8 (3.3–4.4)	0.9 (0.8–1)	0.3 (0.3–0.4)	7.1 (6–8.4)	1.5 (1.3–1.8)
Seropositive rate (%)	0.0 (0.0–0.9)	78.9 (72.7–81.2)	45.1 (39.9–50.3)	0.0 (0.0–1.8)	93.8 (88.8–97.0)	72.1 (64.3–79.0)
Seroconversion rate (%)	N.A.	85.5 (81.6–88.9)	64.8 (59.7–69.7)	N.A.	93.8 (88.8–97.0)	55.2 (47.0–63.2)
GMFI	N.A.	28.2 (23.8–33.3)	6.3 (5.4–7.5)	N.A.	22.5 (18.7–27.2)	4.8 (3.9–6.0)
Anti-S&N-IgG						
GMC (AU/mL)	0.3 (0.2–0.3)	15.0 (13.1–17.2)	3.4 (3–3.9)	0.3 (0.2–0.3)	31.2 (26.4–36.7)	8.5 (7.1–10.1)
Seropositive rate (%)	0.0 (0.0–0.9)	68.6 (63.8–73.2)	19.7 (15.7–24.1)	0.0 (0.0–1.9)	89.4 (83.6–93.7)	45.8 (37.8–54.0)
Seroconversion rate (%)	N.A.	94.1 (91.2–96.2)	81.6 (77.3–85.5)	N.A.	98.1 (94.7–99.6)	94.2 (89.3–97.3)
GMFI	N.A.	59.5 (51.1–69.2)	13.7 (11.9–15.7)	N.A.	116.5 (95.0–142.8)	32.2 (26.2–39.4)

^*a*^ Data are presented as geometric mean (95% CI) or n (%). Alternatively, data are presented as median (IQR) or n/N (%). NAbs positivity was defined as a value exceeding 1:8. A concentration of ≥10.0 AU/mL was considered positive (or reactive) for anti-S&N IgG; a concentration of ≥1.0 AU/mL was considered positive for anti-RBD IgG. Seroconversion was defined as a fourfold increase in concentration after immunization compared with pre-immunization levels. GMT, geometric mean titer; GMC, geometric mean concentration; GMFI, geometric mean fold increase; N.A., not applicable.

We compared IFN-γ and TNF-α levels to evaluate SARS-CoV-2-specific T cell responses in PLWH and HCs (Fig. S1). The level of IFN-γ in PLWH was 9.8 (IQR: 8.4, 14.7) pg/mL, which was lower than that in HCs (46.4 pg/mL, IQR: 12.7, 94.1) at 28 days after the second dose of inactivated COVID-19 vaccine. The level of TNF-α did not differ significantly between the two groups (61.8 pg/mL, IQR: 12.1, 220.7 vs 95.3 pg/mL, IQR: 11.8, 305.0, *P* = 0.943).

Antibody levels declined by 3–6 months after the second dose (T3) in both groups. In PLWH, the GMTs of NAbs, as well as the GMCs of anti-RBD and anti-S&N IgG, were 24.5 (95% CI 22.7–26.5), 0.9 (95% CI 0.8–1.0), and 3.4 (95% CI 3.0–3.9), respectively. These levels were significantly lower than those observed in HCs (32.5 [95% CI 28.8–36.6], 1.5 [95% CI 1.3–1.8], and 8.5 [95% CI 7.1–10.1], respectively). Whereas the seropositivity rate of NAbs in PLWH remained high at 95.1% (95% CI 92.3–97.1) at T3, the seropositivity rates for anti-RBD and anti-S&N IgG decreased to 45.1% (95% CI 39.9–50.3) and 19.7% (95% CI 15.7–24.1), respectively, both lower than in HCs (72.1% [95% CI 64.3–79.0] and 45.8% [95% CI 37.8–54.0]).

### Factors associated with antibody levels

A chi-square and multivariable logistic regression analysis in PLWH assessed the association between the seropositivity rates of NAbs, anti-RBD IgG, and anti-S&N IgG (the main independent variables) and factors such as sex, age, body mass index, years since starting ART, and CD4 counts (Table 4). Age and CD4 levels were the primary factors influencing seropositivity (*P* < 0.01). Compared with those aged 18–39 years, PLWH aged ≥60 years had lower seropositivity rates for NAbs (relative risk [RR] = 0.05; 95% CI, 0.01–0.51), anti-RBD IgG (RR = 0.31; 95% CI, 0.14–0.66), and anti-S&N IgG (RR = 0.43; 95% CI, 0.21–0.85). No significant differences were observed in the 40–59 years age group ([NAbs RR = 0.22; 95% CI, 0.02–2.34], [anti-RBD IgG RR = 0.31; 95% CI, 0.14–0.66], and [anti-S&N IgG RR = 0.31; 95% CI, 0.14–0.66]). Additionally, compared PLWH with the CD4 count <200 cells/µL, PLWH with CD4 count (200–500 cells/µL) and CD4 count ≥500 cells/µL presented a stronger immune response of anti-RBD (RR = 3.39; 95% CI, 1.75–6.57) and (RR = 12.86; 95% CI, 5.23–31.60), anti-S&N IgG (RR = 3.15; 95% CI, 1.65–6.01) and (RR = 12.86; 95% CI, 5.23–31.60).

**TABLE 4:** Multifactor analysis of antibody seropositivity rates^*a*^

Variable	Anti-RBD-IgG				Anti-S&N-IgG				Neutralizing antibody			
	Seropositivity rates (%)	Chi-squared test	Logistic test		Seropositivity rates (%)	Chi-squared test	Logistic test		Seropositivity rates (%)	Chi-squared test	Logistic test	
		*P* value	RR	95% CI		*P* value	RR	95% CI		*P* value	RR	95% CI
Sex		0.372				0.707				1.000^*a*^		
Male	286 (79.4)		Ref		248 (68.9)		Ref		352 (97.8)		Ref	
Female	21 (72.4)		0.76	(0.30–1.98)	19 (65.5)		0.95	(0.40–2.25)	29 (100.0)		N.A.^*c*^	N.A.
Age, years		<0.001^*b*^				0.008^*b*^				0.003^*a*^^*,*^*^b^*		
18–39	193 (84.6)		Ref		166 (72.8)		Ref		227 (99.6)		Ref	
40–59	85 (75.2)		0.62	(0.33–1.16)	77 (68.1)		0.92	(0.54–1.58)	110 (97.3)		0.22	(0.02–2.34)
≥60	29 (60.4)		0.31	(0.14–0.66)	24 (50.0)		0.43	(0.21–0.85)	44 (91.7)		0.05	(0.01–0.51)
Body mass index (kg/m^2^)		0.860				0.813				0.676^*a*^		
≤18.4	28 (80.0)		Ref		23 (65.7)		Ref		35 (100.0)		Ref	
18.5– 24.9	234 (79.3)		1.19	(0.46–3.05)	205 (69.5)		1.35	(0.62–2.96)	289 (98.0)		N.A.	N.A.
≥25.0	45 (76.3)		0.74	(0.24–2.28)	39 (66.1)		0.91	(0.36–2.34)	57 (96.6)		N.A	N.A.
Years of starting ART^c^		0.271				0.607				0.815^*a*^		
<2	77 (74.0)		Ref		68 (65.4)		Ref		101 (97.1)		Ref	
2–4	75 (78.1)		1.14	(0.55–2.36)	65 (67.7)		0.99	(0.53–1.89)	94 (97.9)		1.31	(0.18–9.30)
≥4	155 (82.0)		1.31	(0.67–2.56)	134 (70.9)		1.01	(0.56–1.80)	186 (98.4)		1.81	(0.29–11.5)
CD4 counts (cells/µL)		<0.001^*b*^				<0.001^*b*^				0.007^*a*^^*,^b^*^		
<200	27 (48.2)		Ref		27 (48.2)		Ref		52 (92.9)		Ref	
200–500	155 (77.9)		3.39	(1.75–6.57)	134 (67.3)		3.15	(1.65–6.01)	195 (98.0)		2.64	(0.57–12.33)
≥500	125 (93.3)		12.86	(5.23–31.60)	111 (82.8)		7.12	(3.40–14.93)	134 (100.0)		N.A.	N.A.

^*a*^ Comparisons were performed using the chi-squared test unless otherwise specified as Fisher's exact test. Binary logistic regression was used for multifactorial analysis. Data are presented as relative risks (RR) with 95% confidence intervals.

^*b*^ *P* < 0.05.

^*c*^ N.A, not applicable.

**Fig 3:**
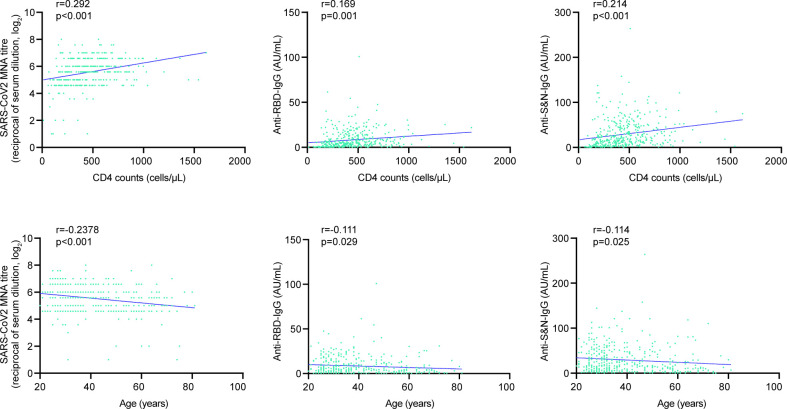
Correlations of antibody levels with CD4+ T cell counts and age. Correlations between antibody levels (NAbs, anti-RBD IgG, and anti-S&N IgG) and CD4+ T cell counts (top) or age (bottom) at T1. Correlations were assessed using Pearson’s correlation coefficient.

Quantitative results were consistent with seropositivity rates. Correlation analysis revealed negative correlations between age and NAbs titers (r = −0.238, *P* < 0.001), anti-RBD IgG (r = −0.111, *P* = 0.029), and anti-S&N IgG (r = −0.114, *P* = 0.025). Positive correlations were observed between CD4 counts and NAbs titers (r = 0.292, *P* < 0.001), anti-RBD IgG (r = 0.169, *P* = 0.001), and anti-S&N IgG (r = 0.214, *P* < 0.001) among PLWH (Fig. 3). Multiple linear regression analysis confirmed significant associations between age and NAbs (B = −0.30, *P* < 0.001), and between CD4 counts and NAbs (B = 0.44, *P* < 0.001), anti-RBD IgG (B = 2.27, *P* = 0.006), and anti-S&N IgG (B = 0.917, *P* < 0.001). Sex, body mass index, and years since starting ART were not associated with NAbs or IgG levels in PLWH (Table 5).

**TABLE 5:** Multiple linear regression analysis of antibody levels^*a*^

Variable	Neutralizing antibody					Anti-RBD-IgG					Anti-S&N-IgG				
	GMT (95% CI)	One-way ANOVA	Multiple linear regression			GMC (95% CI)	One-way ANOVA	Multiple linear regression			GMC (95% CI)	One-way ANOVA	Multiple linear regression		
		*P* value	β	95% CI	*P* value		*P* value	β	95% CI	*P* value		*P* value	β	95% CI	*P* value
Sex		0.851	0.20	(−1.78, 0.57)	0.299		0.57	−0.12	(−4.07, 3.82)	0.951		0.985	4.22	(−7.46, 15.90)	0.478
Male	46.6 (43.2–50.3)					3.9 (3.4–4.9)					15.0 (13.1–17.3)				
Female	50.1 (38.7–64.9)					3.2 (1.8–5.8)					14.9 (8.7–25.7)				
Age, years		<0.001^*b*^	−0.30	(−0.44, −0.15)	<0.001^*b*^		0.002^*b*^	−1.03	(−2.56, 0.50)	0.187		0.003^*b*^	−3.11	(−7.65, 1.43)	0.179
18–39	53.2 (48.8–57.9)					4.6 (3.9–5.5)					17.8 (15.2–20.9)				
40–59	42.7 (37.3–48.9)					3.4 (2.6–4.5)					13.9 (10.6–18.1)				
≥60	32.2 (25.0–41.5)					2.0 (1.3–3.2)					8.2 (5.2–12.8)				
Body mass index (kg/m^2^)		0.535	−0.08	(−0.28, 0.12)	0.430		0.938	1.76	(−0.37, 3.88)	0.105		0.883	5.42	(−0.86, 11.67)	0.090
≤18.4	53.1 (43.4–64.9)					4.0 (2.5–6.4)					14.3 (9.1–22.4)				
18.5–24.9	46.2 (42.6–50.1)					3.8 (3.2–4.4)					15.0 (12.9–17.5)				
≥25.0	47.0 (37.2–59.3)					4.1 (2.8–6.1)					15.7 (10.5–23.4)				
Years of starting ART		0.642	0.03	(−0.10, 0.15)	0.687		0.577	−0.04	(−1.33, 1.24)	0.950		0.399	−0.99	(−4.80, 2.81)	0.607
<2	43.7 (37.5–51.0)					3.3 (2.4–4.4)					11.9 (8.8–16.2)				
2–4	49.2 (42.5–56.9)					4.2 (3.1–5.6)					16.9 (12.7–22.3)				
≥4	47.6 (43.1–52.5)					4.0 (3.3–4.9)					16.1 (13.5–19.2)				
CD4 counts (cells/µL)		<0.001^*b*^	0.44	(0.29, 0.60)	<0.001^*b*^		<0.001^*b*^	2.27	(0.67, 3.87)	0.006		<0.001^*b*^	9.17	(4.42–13.91)	<0.001^*b*^
<200	29.3 (22.9–37.5)					1.6 (1.0–2.4)					5.5 (3.5–8.7)				
200–500	45.3 (41.1–49.9)					3.6 (2.9–4.4)					14.2 (11.9–17)				
≥500	60.1 (54.6–66.2)					6.2 (5.1–7.5)					24.8 (20.9–29.5)				

^*a*^ One-way analysis of variance with Tukey's multiple comparisons test to analyze log-transformed antibody titers and categorical data, Wilcoxon rank-sum test was utilized for non-normally distributed data. Multiple linear regression was conducted to evaluate independent risk factors.

^*b*^ *P* < 0.05.

### Low immune response to Covilo booster

**Fig 4:**
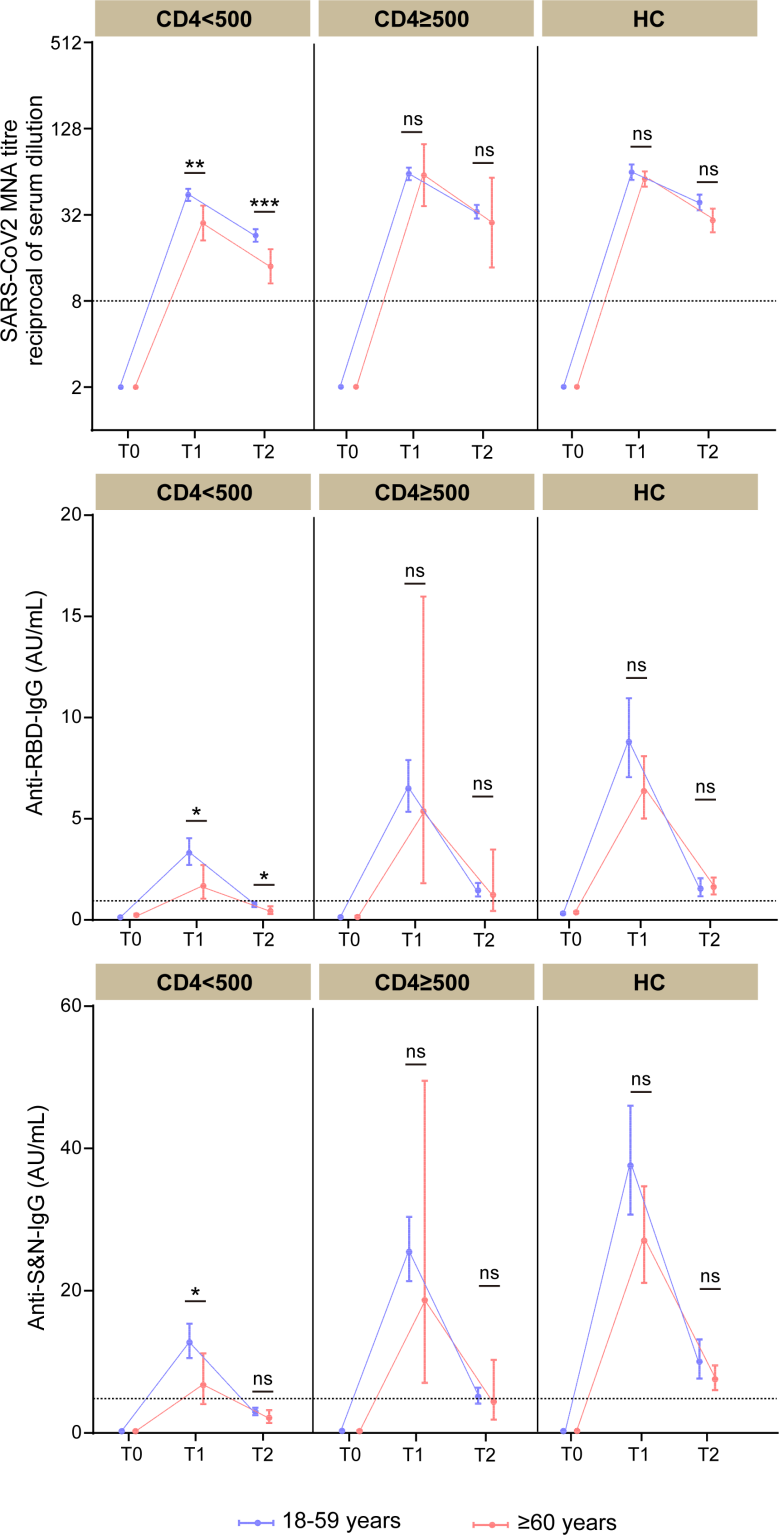
Dynamic changes in antibody levels across different CD4+ T cell groups. Titers of NAbs, anti-RBD IgG, and anti-S&N IgG were measured at baseline (T0), 4 weeks (T1), and 12 weeks (T2) after the second dose of Covilo in PLWH with CD4+ T cell counts <500 cells/µL or ≥500 cells/µL, and in HCs. Data are presented as geometric mean ± 95% confidence interval (CI). Statistical significance was determined using the Mann–Whitney U test. *P* values <0.05 were considered statistically significant. 95% CI, 95% confidence interval; MNA, microneutralization assays. ns, not significant; *P < 0.05, **P < 0.01, ***P < 0.001, ****P < 0.0001.

Twenty-eight days after the second Covilo dose, antibody levels in participants aged 18–59 and ≥60 years with CD4 counts ≥500 cells/µL did not significantly differ between PLWH and HCs (Fig. 3). However, among PLWH with CD4 counts <500 cells/µL, the ≥60-year-old group had significantly lower antibody levels than the 18–59-year-old group. This was observed for NAbs (28.0 [95% CI 21.2–37.1] vs 44.1 [95% CI 40.0–48.7]), anti-RBD IgG (1.7 [95% CI 1.0–2.7] vs 3.3 [95% CI 2.7–4.0]), and anti-S&N IgG (6.8 [95% CI 4.1–11.2] vs 12.7 [95% CI 10.6–15.4]). Similar results were observed at T2: 14.0 (95% CI 10.6–18.4) vs 22.9 (95% CI 20.7–25.4) for NAbs, 0.4 (95% CI 0.3–0.7) vs 0.8 (95% CI 0.6–0.9) for anti-RBD IgG, and 2.1 (95% CI 1.4–3.2) vs 3.0 (95% CI 2.5–3.5) for anti-S&N IgG (Fig. 4).

**Fig 5:**
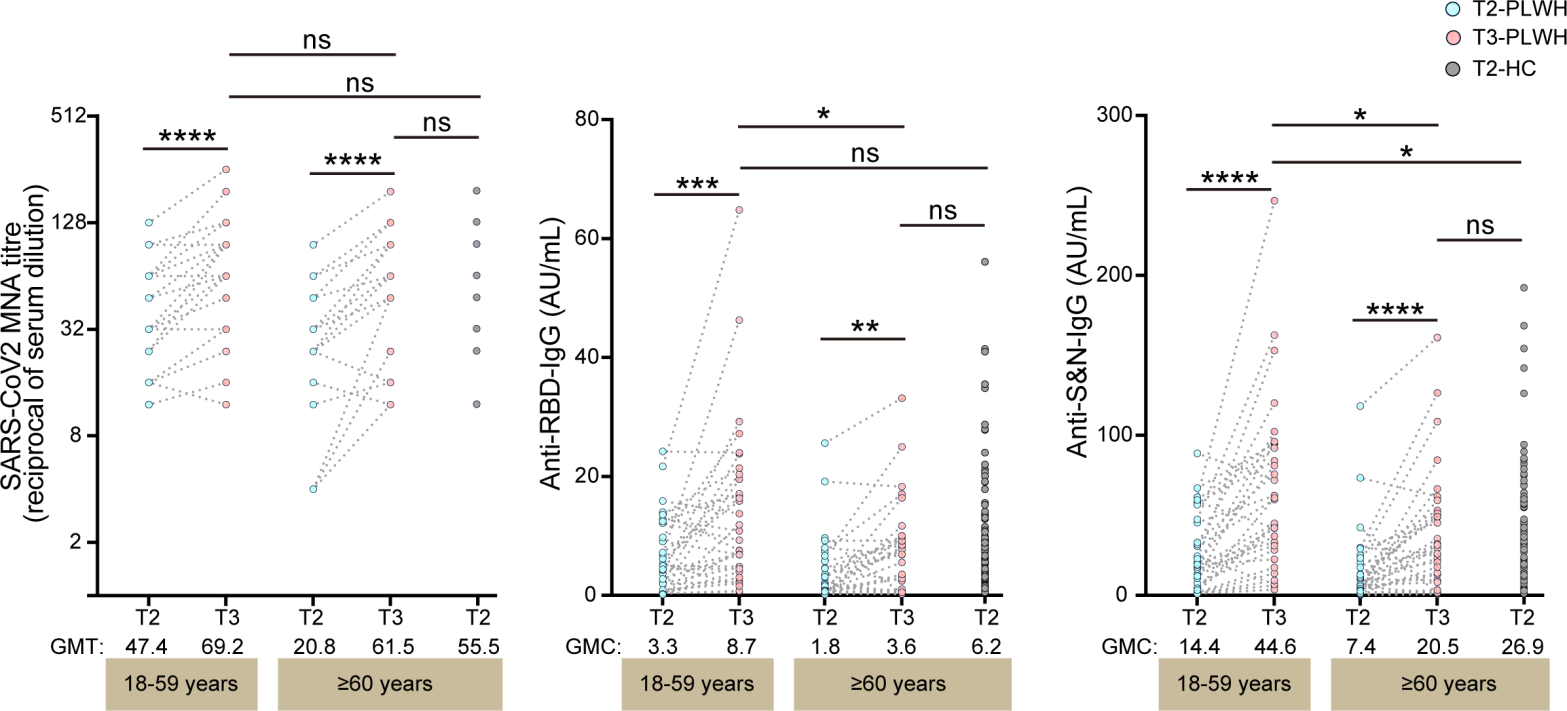
Antibody responses after a Covilo booster in PLWH with CD4+ T cell counts <500 cells/µL. PLWH with CD4+ T cell counts <500 cells/µL (aged 18–59 and ≥60 years, *n* = 32) received a booster dose of Covilo. Titers of NAbs, anti-RBD IgG, and anti-S&N IgG were measured 28 days after the booster (T3). Data are presented as geometric mean ± 95% CI. Statistical significance was determined using the Mann–Whitney U test and the Wilcoxon rank-sum test. *P* values <0.05 were considered statistically significant. 95% CI, 95% confidence interval; MNA, microneutralization assays. ns, not significant; *P < 0.05, **P < 0.01, ***P < 0.001, ****P < 0.0001.

Twenty-eight days after receiving a third dose of Covilo (T3), the GMTs of NAbs in PLWH with CD4 counts <500 cells/µL and aged ≥60 years increased to levels comparable to those observed in HCs aged ≥60 years after their second dose (GMT 61.5 [95% CI 47.8–79.2] vs 55.5 [95% CI 49.1–62.6]) (Fig. 5). A similar trend was observed for the GMCs of anti-RBD IgG (3.6 [95% CI 2.1–6.1] vs 6.2 [95% CI 4.8–7.8]) and anti-S&N IgG (20.5 [95% CI 12.2–34.5] vs 26.9 [95% CI 20.1–34.5]). However, in PLWH aged 18–59 with CD4 counts <500 cells/µL, anti-RBD IgG and anti-S&N IgG levels were significantly increased at T3 (44.6 [95% CI 31.0–64.0]). These findings emphasize the challenges PLWH with low CD4 counts and advanced age face in achieving a robust immune response to the inactivated COVID-19 vaccine. Additionally, we found that the second and third doses of inactivated COVID-19 vaccine failed to elicit a perfect neutralizing antibody response to the Omicron variants (e.g., XBB.1.5 and EG.5) in PLWH (Fig. S2).

## DISCUSSION

The COVID-19 pandemic continues to pose a threat to global public health, despite the World Health Organization declaration that COVID-19 is no longer a Public Health Emergency of International Concern (31). Recent studies have revealed that immunocompromised populations, such as PLWH, remain at higher risk for severe COVID-19 illness and mortality; their immune response to COVID-19 vaccines may also be weaker than that of healthy individuals (5, 21). An understanding of this response is crucial for efforts to develop additional measures that can prevent future outbreaks. This prospective study examined the safety and immunogenicity of a two-dose regimen of the inactivated COVID-19 vaccine (Covilo) in PLWH, then investigated factors associated with antibody levels. Our findings contribute to the growing body of evidence regarding the inadequate immune response in PLWH, particularly among older individuals and those with low CD4+ T cell counts.

There is evidence that inactivated COVID-19 vaccines are a safe option for special populations (32). Here, we reported the incidence of adverse reactions within 7 days after the first and second doses of Covilo in both PLWH and HCs. Similar to previous findings, adverse reactions were mild or self-limiting in the present study (21, 33, 34). Approximately 12% of PLWH experienced adverse reactions after vaccination, with no serious adverse events reported. Injection site pain was the most common adverse reaction in both PLWH and HCs after each dose. Although the incidence of adverse events was higher in PLWH than in HCs, it was lower than the incidence reported for other vaccine types (35–37). This discrepancy may be due to several factors, including decreased sensitivity to discomfort in PLWH due to underlying health conditions and the negative effects of long-term antiviral medications. Overall, inactivated COVID-19 vaccination appears to be relatively well-tolerated in PLWH.

Previous studies showed that the levels of NAbs and SARS-CoV-2-specific antibodies were lower and declined more rapidly in PLWH than in HCs after receipt of inactivated COVID-19 vaccines (38, 39). Similarly, we found that PLWH had a weaker immune response to Covilo after the second dose, characterized by lower titers of NAbs and lower concentrations of anti-RBD IgG and anti-S&N IgG. These results are consistent with the observation that HIV infection is associated with decreased antibody responses to other vaccines, such as the influenza vaccine (13, 40). However, a study conducted in South Africa and the United Kingdom showed that serological responses to the ChAdOx1 nCoV-19 vaccine were similar in PLWH and HCs. The same study revealed no significant differences in the magnitude or persistence of SARS-CoV-2 spike-specific humoral or cellular responses between PLWH and HCs after ChAdOx1 vaccination (41). This discrepancy may be attributed to differences in vaccine types and the health status of PLWH, including the effectiveness of ART. In our study, both NAbs and IgG levels declined over time in PLWH and HCs. Notably, whereas the seropositivity rates for anti-RBD and anti-S&N IgG in PLWH decreased to 45.1% and 19.7%, respectively, the seropositivity rate for NAbs remained high at 95.1%, 3–6 months after the second dose of Covilo. This decline in antibody concentration raises concerns about the duration of immune protection induced by vaccination.

Epidemiological studies have demonstrated that various risk factors in PLWH can influence serological responses to both convalescent serum samples and COVID-19 vaccines (42, 43). The CD4+ T cell count is considered an independent predictor of discordant immune responses among PLWH; it is crucial for inducing humoral immune responses to mRNA, adenovirus vector, and inactivated COVID-19 vaccines (14, 44, 45). In PLWH, particularly those with low CD4+ T cell counts, immune senescence and chronic inflammation impair T cell function and B cell activation. These factors disrupt germinal center reactions, resulting in weaker and shorter-lived antibody responses to inactivated COVID-19 vaccines. Our study also showed that PLWH with CD4+ T cell counts <500 cells/µL developed a weaker and less durable immune response after two doses of an inactivated COVID-19 vaccine. The CD4 count has been linked to decreased humoral responses to several vaccines, including hepatitis A, hepatitis B, and pneumococcal vaccines, in PWLH (46–48). Additionally, our correlation and multifactor analyses revealed that age (≥60 years) was also associated with a weaker immune response after vaccination. Therefore, special consideration is needed when vaccinating older PLWH with low nadir CD4+ T cell counts; such individuals may struggle to achieve adequate immunity even after a booster dose. These findings highlight a critical challenge and emphasize the importance of promoting COVID-19 vaccine uptake and overall immunization in this vulnerable population.

The observation that the second and third doses of inactivated COVID-19 vaccines failed to induce a perfect neutralizing antibody response against XBB.1.5 and EG.5 in PLWH highlights a key limitation in vaccine performance. This is particularly concerning in immunocompromised populations, who may already exhibit diminished immune responses to vaccination. As SARS-CoV-2 continues to evolve, the emergence of antigenically distinct variants, such as XBB.1.5 and EG.5, underscores the importance of developing vaccines that can elicit broader and more durable immune responses. Updating vaccines to better target Omicron and its subvariants would not only enhance protection in vulnerable populations but also improve global vaccine effectiveness.

This prospective study has several limitations. First, given the numerous reports on the safety and immunogenicity of inactivated COVID-19 vaccines in PLWH, our study offers limited innovation in this context. The primary aim of this study was to highlight the vulnerability of older PLWH with low nadir CD4+ T cell counts; these individuals may be immunological non-responders to multiple vaccines. Second, the age/sex distribution between the PLWH and healthy control groups in your study was imbalanced. A previous study showed that Covilo elicited similar responses in males and females, which may mitigate the effect of gender imbalance on our results (49). Others and our previous study have shown that the elderly population induces lower antibody levels (24, 27), although PLWH with a lower elderly population still induced lower antibody levels in this study, reflecting the fact that the low immune response of PLWH has a greater impact on the vaccine. Third, due to the rapidly changing COVID-19 vaccination policies and control measures in China, we were unable to extend the follow-up period to 12–18 months for long-term immunity in PLWH. Finally, while our study assessed IFN-γ and TNF-α levels as indicators of T-cell responses, the lack of comprehensive phenotyping (e.g., memory subsets, activation/exhaustion markers) precludes deeper mechanistic insights. Future work should investigate these parameters to elucidate immune regulation in older PLWH following inactivated COVID-19 vaccination.

In conclusion, our study demonstrates that PLWH exhibit weaker immune responses to inactivated COVID-19 vaccines compared to healthy controls, particularly in terms of antibody levels and cytokine production. These findings underscore the necessity for targeted vaccination strategies for PLWH, especially older adults, to mitigate their unique immunological challenges. Prioritizing booster doses and exploring alternative vaccine platforms could enhance protection against COVID-19 and reduce the risk of severe outcomes in this vulnerable population.

However, the short follow-up period (up to 6 months) in our study limits our ability to draw definitive conclusions about the longevity of vaccine-induced immune responses in PLWH. Future studies with longer follow-up durations are needed to evaluate the durability of these responses and refine vaccination strategies for immunocompromised individuals. Despite this limitation, our findings contribute valuable insights toward developing more equitable and effective vaccination approaches for PLWH and other immunocompromised populations.

## Data Availability

The data that support the findings of this study are available from the corresponding author upon reasonable request.

## SUPPLEMENTAL MATERIAL

Supplemental material jvi.00688-25-s0001.docx Figures S1 to S3, Table S1, and supplemental methods.

ASM does not own the copyrights to Supplemental Material that may be linked to, or accessed through, an article. The authors have granted ASM a non-exclusive, world-wide license to publish the Supplemental Material files. Please contact the corresponding author directly for reuse.
