# Supplementary material for: Inadequate immune response to inactivated COVID-19 vaccine among older people living with HIV: a prospective cohort study
Source: J Virol. 2025 Aug 21;99(9):e00688-25. doi: 10.1128/jvi.00688-25 (PMC12456149; doi:10.1128/jvi.00688-25)
Supplement: Supplemental material — Figures S1 to S3, Table S1, and supplemental methods. [file jvi.00688-25-s0001.docx]

**Supplemental material for publication**

**
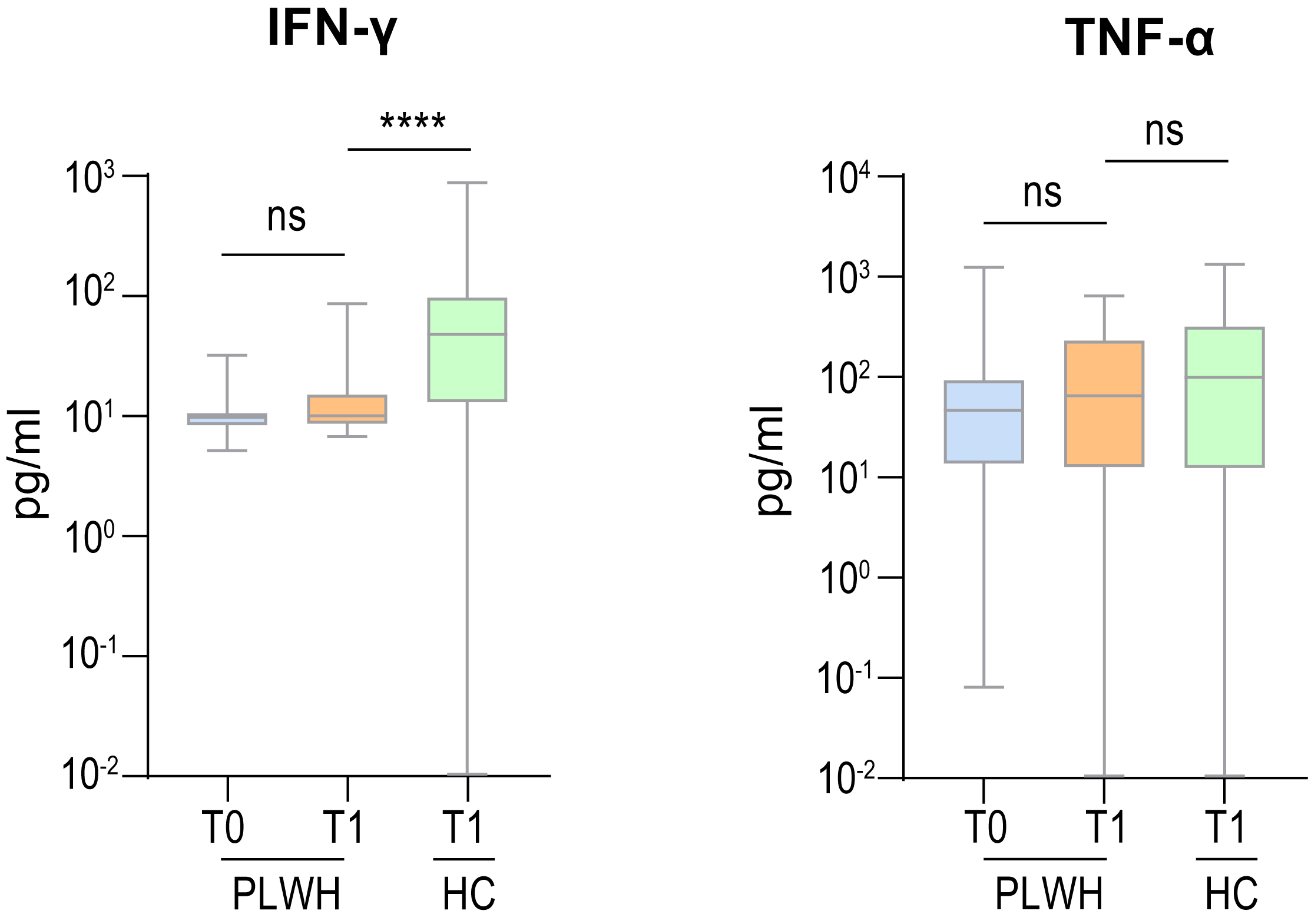
**

**Fig S1. SARS-CoV-2-specific T cell responses.** PBMCs were collected at baseline (T0) and 28 days (T1) after the second dose of Covilo in PLWH and HCs. The supernatant of PBMCs stimulated by peptide library (S protein of wide type SARS-CoV-2 ) and detected by IFN-γ and TNF-α ELISA Kit. Statistical significance was determined using the Mann–Whitney U test and the Wilcoxon rank-sum test. P-values <0.05 were considered statistically significant. IQR: interquartile range, MNA: microneutralization assays. ns, not significant.

**
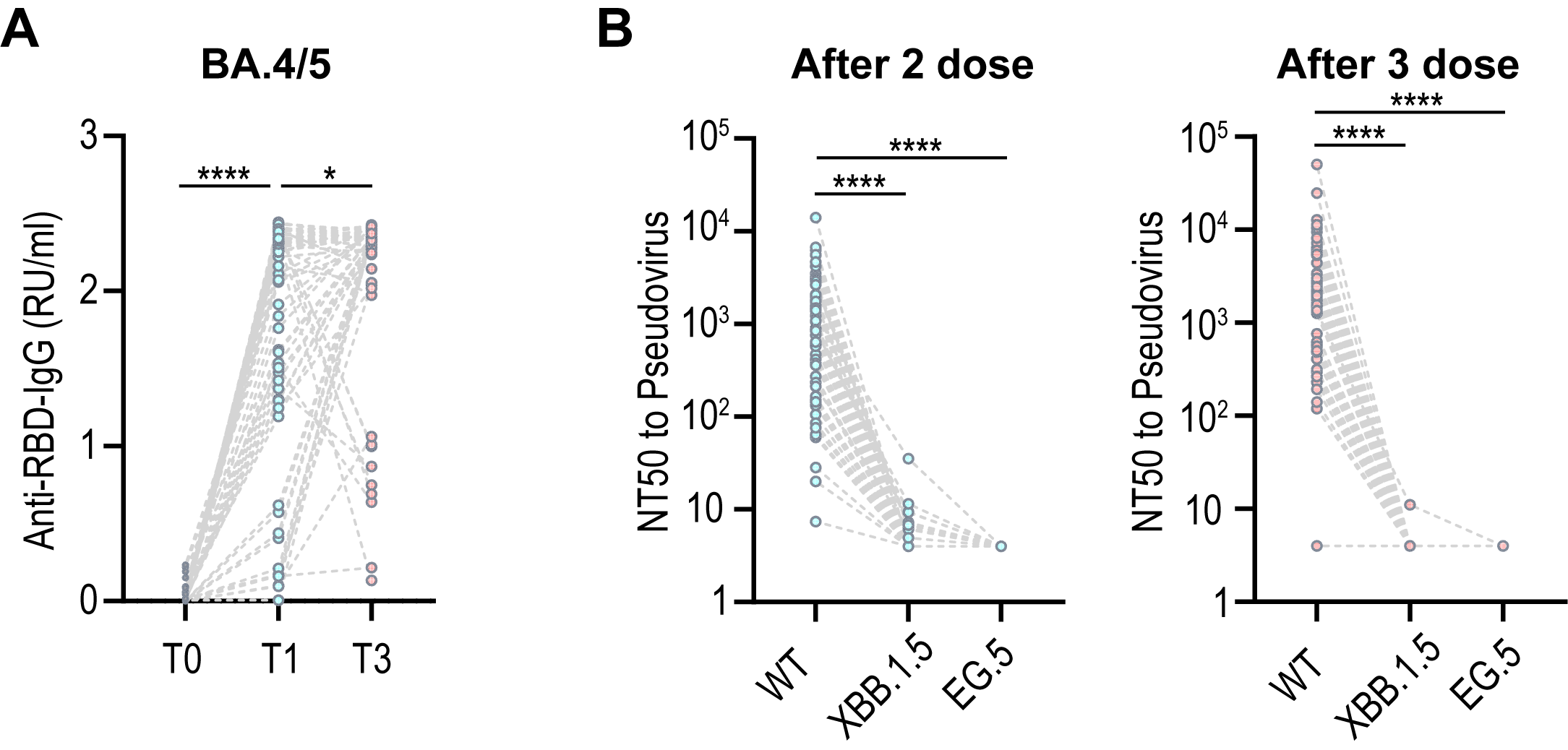
**

**Fig.S2 Antibody against Omicron variants. (A)** anti-RBD IgG of BA.4/5 and **(B)** NT50 of pseudovirus against Omicron variants (BA.4/5, XBB.1.5, and EG.5) at baseline (T0) and 28 days (T1) after the second dose of Covilo, 28 days after the booster (T3) in PLWH. Statistical significance was determined using the Mann–Whitney U test and the Wilcoxon rank-sum test. P-values <0.05 were considered statistically significant. 95% CI: 95% confidence interval, MNA: microneutralization assays. ns, not significant; *P<0.05, **P<0.01, ***P<0.001, ****P<0.0001.

**
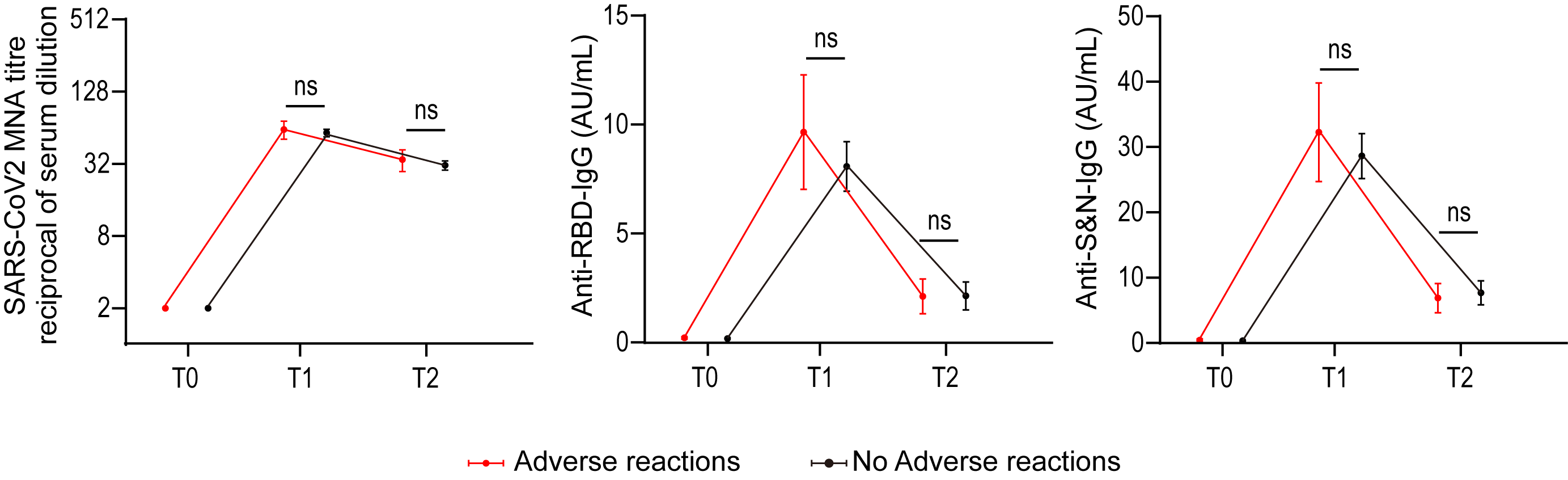
**

**Fig.S3 Influence of adverse reaction on antibody levels.** Titers of NAbs, anti-RBD IgG, and anti-S&N IgG in adverse reaction or no adverse reaction groups at baseline (T0), 28 days (T1), and 3–6 months (T2) after the second dose of Covilo in PLWH. Statistical significance was determined using the Mann–Whitney U test and the Wilcoxon rank-sum test. P-values <0.05 were considered statistically significant. 95% CI: 95% confidence interval, MNA: microneutralization assays. ns, not significant; *P<0.05, **P<0.01, ***P<0.001, ****P<0.0001.

**Table S1. Adverse reactions within 7 days by age.**

| **Variable** | **PLWH (n=400)** | | **HCs(n=190)** | |
| --- | --- | --- | --- | --- |
|  | 18-59 years old | ≥60 years old | 18-59 years old | ≥60 years old |
| **First dose** |  |  |  |  |
| Total adverse reactions | 46(13.1) | 2(4.1) | 2(2.4) | 3(2.8) |
| Local reactions | 21(6.0) | 1(2.0) | 1(1.2) | 2(1.9) |
| Itching | 1(0.3) | 0(0) | 0(0) | 0(0) |
| Pain | 15(4.3) | 1(2.0) | 1(1.2) | 1(0.9) |
| Induration | 0(0) | 0(0) | 0(0) | 0(0) |
| Redness | 0(0) | 0(0) | 0(0) | 0(0) |
| Swelling | 1(0.3) | 0(0) | 0(0) | 1(0.9) |
| Rash | 5(1.4) | 0(0) | 0(0) | 0(0) |
| Systemic reactions | 32(9.1) | 1(2.0) | 1(1.2) | 1(0.9) |
| Coughing | 1(0.3) | 0(0) | 0(0) | 0(0) |
| Diarrhea | 7(2.0) | 0(0) | 0(0) | 0(0) |
| Fatigue | 8(2.3) | 0(0) | 0(0) | 0(0) |
| Fever | 2(0.6) | 0(0) | 0(0) | 0(0) |
| Headache | 11(3.1) | 0(0) | 1(1.2) | 0(0) |
| Myalgia | 9(2.6) | 1(2.0) | 0(0) | 0(0) |
| Nausea and vomiting | 5(1.4) | 0(0) | 0(0) | 1(0.9) |
| **Second dose** |  |  |  |  |
| Total adverse reactions | 21(6.0) | 1(2.1) | 0(0) | 4(3.7) |
| Local reactions | 16(4.6) | 0 | 0(0) | 2(1.9) |
| Itching | 0(0) | 0(0) | 0(0) | 0(0) |
| Pain | 15(4.3) | 0(0) | 0(0) | 2(1.9) |
| Induration | 0(0) | 0(0) | 0(0) | 0(0) |
| Redness | 0(0) | 0(0) | 0(0) | 0(0) |
| Swelling | 1(0.3) | 0(0) | 0(0) | 0(0) |
| Rash | 2(0.6) | 0(0) | 0(0) | 0(0) |
| Systemic reactions | 9(2.6) | 1(2.1) | 0(0) | 3(2.8) |
| Coughing | 1(0.3) | 0(0) | 0(0) | 0(0) |
| Diarrhea | 1(0.3) | 0(0) | 0(0) | 0(0) |
| Fatigue | 6(1.7) | 1(2.1) | 0(0) | 3(2.8) |
| Fever | 0(0) | 0(0) | 0(0) | 0(0) |
| Headache | 0(0) | 0(0) | 0(0) | 0(0) |
| Myalgia | 1(0.3) | 0(0) | 0(0) | 0(0) |
| Nausea and vomiting | 0(0) | 0(0) | 0(0) | 0(0) |

Data are presented as the number of participants experiencing the event (%). Each participant was counted only once within each specific reaction category, even if they experienced multiple adverse reactions. The "Total" category includes all participants who experienced adverse reactions or events of any grade. Adverse reactions and events were graded according to the scale issued by the China State Food and Drug Administration.

**Supplemental Methods**

**1. Sample Preparation of PBMCs**

Whole blood was collected in ethylenediaminetetraacetic acid (EDTA)-containing tubes (BD Biosciences, San Jose, CA, USA) and processed within 12 hours of collection. Peripheral blood mononuclear cells (PBMCs) were isolated from the blood using Ficoll-Hypaque density gradient centrifugation. The isolated PBMCs were stored in liquid nitrogen for future experiments.

1. **Assessment of SARS-CoV-2-Specific T Cell Responses**

We also evaluated SARS-CoV-2–specific T cell responses by measuring IFN-γ and TNF-α production after stimulation with peptide library (S protein of wide type SARS-CoV-2 ) . The ELISA assay for IFN-γ and TNF-α detection was performed using a commercial kit (U-CyTech biosciences). Briefly, cell culture supernatants were collected, clarified, and stored at -80°C. ELISA plates were coated with capture antibody overnight at 4°C, followed by blocking with 1% BSA in PBS. Standards and samples were added in duplicate, and plates were incubated for 2 hours. After washing, biotinylated detection antibody and HRP-conjugated streptavidin were sequentially added, followed by TMB absorbance was measured at 450 nm. IFN-γ and TNF-α concentrations were calculated using a 4-parameter logistic curve.

**3.Neutralizing antibodies against pseudoviruses**

A pseudovirus assay was developed to evaluate the neutralizing antibody potencies of serum samples against SARS-CoV-2 Omicron (Wildtype, XBB.1.5, and EG.5) pseudoviruses. Pseudoviruses were generated by cloning the spike proteins of these strains into the pCAGGS vector. Spike proteins with an 18-amino acid deletion from the C-terminus of the SARS-CoV-2 Wuhan-1 reference strain (GISAID: EPI_ISL_402119) were used for pseudovirus production. Codon optimization ensured compatibility with mammalian cell expression. Plasmid constructs (30 mg each) were transfected into HEK-293T cells, followed by the addition of VSV-DG-GFP pseudotyped virus 24 hours post-transfection. After 2 hours of infection, the medium was replaced with fresh complete DMEM containing anti-VSV-G antibodies (I1-Hybridoma-CRL2700™, ATCC). Incubation continued for 30 hours at 37℃. Supernatants were then collected, filtered through a 0.45-mm filter (Millipore Cat#SLHP033RB), aliquoted, and stored at -80℃. For the neutralization assay, serum samples were initially diluted to 1:8 and then serially diluted two-fold in a 96-well plate. Pseudotyped virus was added to the diluted serum and incubated for 1 hour at 37°C. The mixture was then transferred to a 96-well plate containing Vero cells. After an additional 20 hours of incubation, transducing units (TUs) were quantified using the EVOS M7000 Automated Live Cell Fluorescence Imaging System (Thermo Fisher Scientific) and ImageJ software. The median serum dilution required to neutralize 50% of the virus (NT50) was determined using nonlinear regression with GraphPad Prism software. Geometric mean titers (GMTs) of NT50 titers for SARS-CoV-2 Omicron variants are reported.
